# Optical Coherence Tomography Angiography (OCTA) Characteristics of Acute Retinal Arterial Occlusion: A Systematic Review

**DOI:** 10.3390/healthcare13162056

**Published:** 2025-08-20

**Authors:** Saud Aljohani

**Affiliations:** Department of Ophthalmology, Imam Abdulrahman Bin Faisal University, Dammam 34212, Saudi Arabia; saljohani@iau.edu.sa

**Keywords:** optical coherence tomography angiography (OCTA), retinal arterial occlusion (RAO), central retinal artery occlusion (CRAO), branch retinal artery occlusion (BRAO), superficial capillary plexus (SCP), deep capillary plexus (DCP), vessel density

## Abstract

**Purpose:** To systematically review the evidence regarding the characteristics of Optical Coherence Tomography Angiography (OCTA) in acute retinal arterial occlusion (RAO), with a particular focus on vascular alterations across the superficial and deep capillary plexuses, choroid, and peripapillary regions. **Methods**: A comprehensive literature search was performed across PubMed, Web of Science, Scopus, EMBASE, Google Scholar, and the Cochrane Database up to April 2025. The search terms included “Optical coherence tomography angiography,” “OCTA,” “Retinal arterial occlusion,” “Central retinal artery occlusion,” and “Branch retinal artery occlusion.” Studies were included if they evaluated the role of OCTA in diagnosing or assessing acute RAO. Case reports, conference abstracts, and non-English articles were excluded. Two reviewers independently conducted the study selection and data extraction. The methodological quality of the included studies was assessed using the Risk of Bias in Non-randomized Studies of Interventions (ROBINS-I) tool. **Results**: The initial search yielded 457 articles, from which 10 studies were ultimately included in the final analysis after a rigorous screening process excluding duplicates, non-English publications, and ineligible articles based on title, abstract, or full-text review. The included studies consistently demonstrated that OCTA is a valuable, noninvasive modality for evaluating microvascular changes in RAO. Key OCTA findings in acute RAO include significant perfusion deficits and reduced vessel density in both the superficial capillary plexus (SCP) and deep capillary plexus (DCP). Several studies noted more pronounced involvement of the SCP compared to the DCP. OCTA parameters, such as vessel density in the macular region, have been found to correlate with visual acuity, suggesting a prognostic value. While findings regarding the foveal avascular zone (FAZ) were mixed, the peripapillary area frequently showed reduced vessel density. **Conclusion**: Acute RAO is an ocular emergency that causes microvascular ischemic changes detectable by OCTA. This review establishes OCTA as a significant noninvasive tool for diagnosing, monitoring, and prognosticating RAO. It effectively visualizes perfusion deficits that correlate with clinical outcomes. However, limitations such as susceptibility to motion artifacts, segmentation errors, and the lack of standardized normative data must be considered. Future standardization of OCTA protocols and analysis is essential to enhance its clinical application in managing RAO.

## 1. Introduction

Acute retinal arterial occlusion (RAO) is an ocular emergency characterized by a sudden, painless loss of vision. Clinical examination reveals characteristic features, including opacification of the infarcted ganglion cell layer surrounding the fovea, a cherry-red spot, and, in some cases, a visible central retinal artery (CRA) embolus or other retinal arterial emboli [1,2,3,4]. RAO occurs due to occlusion of blood flow in the main central retinal artery or its tributaries. Depending on the location of the occlusion, RAO can be classified as either central retinal artery occlusion (CRAO) or branch retinal artery occlusion (BRAO) [1,5]. The retina receives oxygen both from the CRA and via passive diffusion of oxygen from the outer retina and choroid [6]. Areas of the inner retina supplied by cilioretinal arteries, which are not branches of the CRA, may retain visual function. If a cilioretinal artery supplies the macula despite surrounding infarction from CRAO, central vision may remain preserved [1,3,4]. RAO is usually diagnosed based on history, clinical examination, and imaging modalities such as fluorescein angiography (FA), optical coherence tomography (OCT), and fundus photography. FA is considered the gold standard for diagnosing RAO due to its ability to visualize retinal perfusion. However, the procedure is time-consuming and requires experienced personnel, which may delay diagnosis and treatment. Furthermore, FA may have limited capability in assessing the deeper retinal capillary plexuses due to light scattering caused by opacification of the inner retinal layers. OCT can be utilized to determine the specific retinal level of edema or to detect subsequent atrophy during the chronic stage, when FA may no longer reveal perfusion deficits [4,6,7]. The period from RAO onset to irreversible inner retinal infarction is recognized as the “golden time” for treatment. Unfortunately, this therapeutic window is very short [1,8,9,10]. Optical coherence tomography angiography (OCTA) is a rapid, noninvasive imaging modality that has significantly improved retinal vascular imaging. It provides dye-free, high-resolution visualization of the retinal and choroidal microvasculature and enables detailed assessment of vascular changes without the need for highly experienced personnel [7,11,12]. In addition, OCTA offers several advantages over other imaging techniques. It facilitates three-dimensional and *en face* visualization of blood flow within the retinal and choroidal vasculature, thereby enabling the detection of flow deficits associated with acute vascular interruption in RAO. It also provides segmented *en face* imaging of the deep vascular plexus, which is poorly visualized with FA, and reveals finer details of the superficial vascular plexus [4,13,14]. Several studies have assessed the role of OCTA in diagnosing retinal arterial occlusion by characterizing perfusion changes and related vascular abnormalities [4,7,12,13,14,15,16,17,18,19]. A systematic review of the current OCTA evidence in RAO can offer valuable insight into its diagnostic utility and limitations. The purpose of this review article is to provide an overview of OCTA features in acute RAO, with an emphasis on its diagnostic utility, observed vascular changes, and clinical implications.

## 2. Materials and Methods

An a priori protocol was prospectively developed and registered in the PROSPERO database (CRD420251073840). Subsequently, the systematic review was conducted in accordance with the Cochrane Handbook for Systematic Reviews of Interventions and the Preferred Reporting Items for Systematic Reviews and Meta-Analyses (PRISMA) guidelines. A comprehensive literature search was performed, with the assistance of an expert librarian, using PubMed, Web of Science, Scopus, EMBASE, Google Scholar, and the Cochrane Database up to April 2025. The search strategy included the keywords: (“Optical coherence tomography angiography” OR “OCTA”) AND (“Retinal arterial occlusion” OR “Central retinal artery occlusion” OR “Branch retinal artery occlusion” OR “RAO” OR “CRAO” OR “BRAO”). Additional references were identified by screening the bibliographies of selected articles. Studies were eligible for inclusion if they focused on the role of OCTA in diagnosing or evaluating acute RAO, including its diagnostic capabilities, vascular characteristics, and clinical implications. Case reports, conference abstracts, and non-English articles were excluded. To ensure methodological rigor and minimize bias, study selection and data extraction were independently performed by two reviewers (SJ, AS). Each reviewer screened titles and abstracts, followed by full-text assessment of potentially eligible studies based on predefined inclusion and exclusion criteria. Data extraction was likewise performed independently. Discrepancies at any stage were resolved through discussion. The complete search terms used for each database are presented in (Table 1).

### Assessment of the Methodological Quality

**Figure 1:**
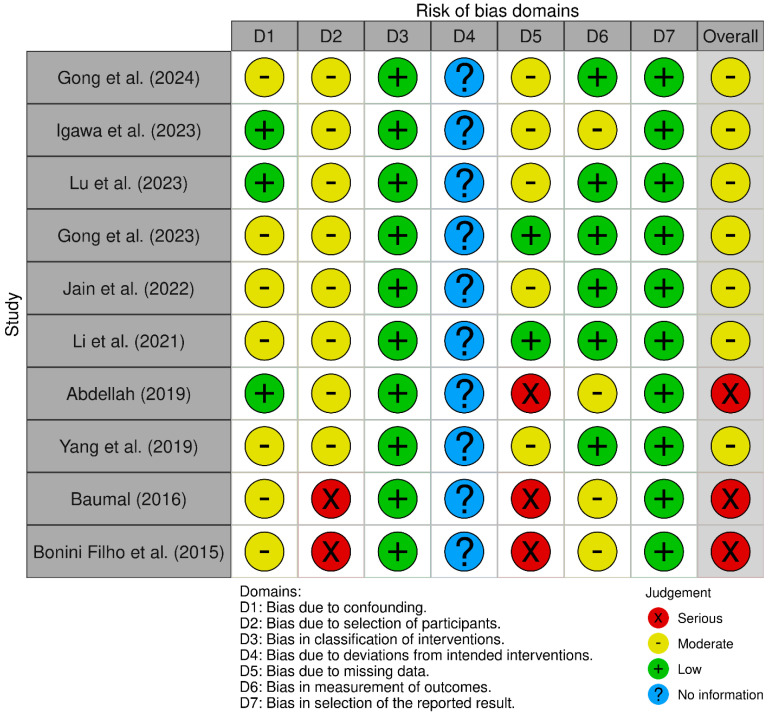
This presents a summary of the proportion of risk of bias assessments across seven methodological domains and the overall risk of bias for the included studies [4,7,12,13,14,15,16,17,18,19]. The highest proportion of low-risk judgments was observed in the domains of classification of interventions and measurement of outcomes, indicating consistent methodological rigor in these areas. Conversely, the domain of deviations from intended interventions exhibited the greatest proportion of unclear risk (no information), suggesting a widespread lack of reporting on adherence to intervention protocols. Serious risk of bias was most commonly observed in the domains related to the selection of participants, missing data, and selective reporting, highlighting persistent concerns regarding participant inclusion criteria, data completeness, and outcome transparency. The overall risk of bias revealed a predominance of moderate and serious concerns, emphasizing the need for improved methodological practices and reporting standards in future studies.

We evaluated the methodological quality of the included studies using the *Risk of Bias in Non-randomised Studies of Interventions* (ROBINS-I) tool [20]. The ROBINS-I tool assesses seven types of bias: bias due to confounding; bias in the selection of study participants; bias in the classification or measurement of interventions or exposures; bias due to deviations from intended interventions or exposures; bias due to missing data; bias in the measurement of outcomes; and bias in the selection of reported results (Table 2). The risk of bias across the included studies is summarized in Figure 1. Each study was evaluated across the seven domains: bias due to confounding (D1); selection of participants (D2); classification of interventions (D3); deviations from intended interventions (D4); missing data (D5); measurement of outcomes (D6); and selection of the reported result (D7). Judgements were categorized as low (green), moderate (yellow), serious (red), or no information available (blue). Most of the recent studies (e.g., Gong et al., [14]; Igawa et al., [13]; Lu et al., [12]) demonstrated a low to moderate risk of bias across most domains. However, consistent uncertainty was observed in D4, indicating insufficient reporting on adherence to intended interventions. In contrast, older studies, such as those by Baumal [4] and Bonini Filho et al. [17], exhibited serious risk in several domains, particularly in participant selection (D2) and missing data (D5), contributing to an overall serious risk classification. These findings highlight variability in methodological rigor, with a trend toward improved reporting quality in more recent research. Nonetheless, the recurrent lack of information in key domains underscores the need for greater transparency and completeness in future studies.

## 3. Results

**Figure 2:**
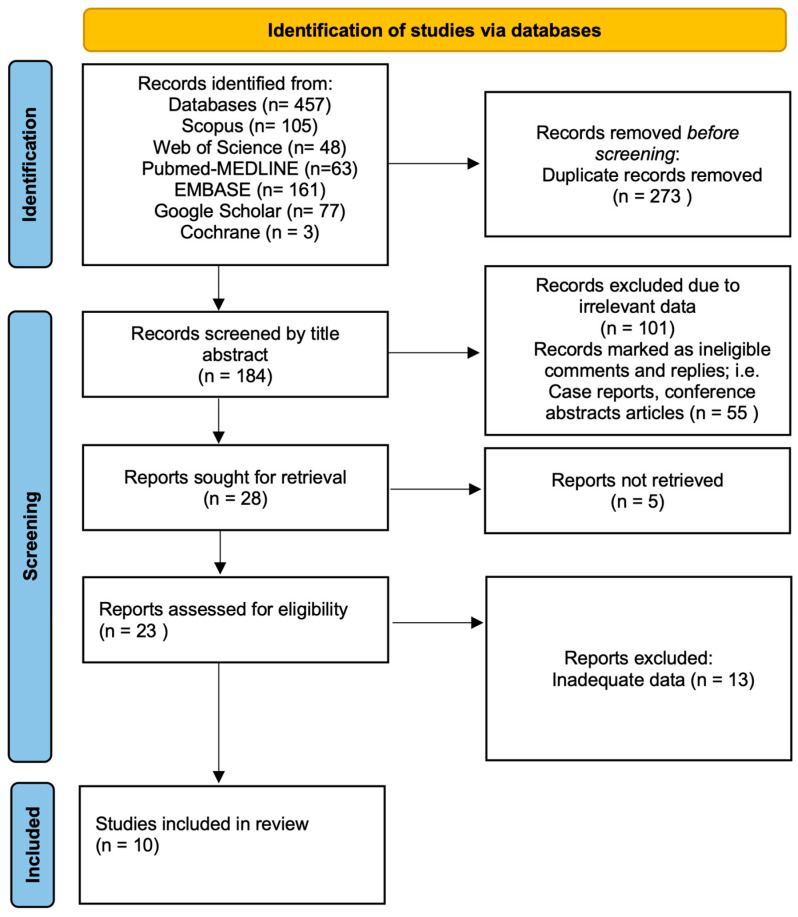
PRISMA flow diagram of literature review process.

A total of 457 articles were initially identified with the assistance of an expert librarian from the Directorate of Library Affairs at our university. Duplicate records were removed using Zotero software (v 7.0.22), resulting in 184 unique articles. These were exported to the Rayyan tool for title and abstract screening, which led to the exclusion of 156 ineligible articles. Of the remaining 28 articles, 5 were excluded due to publication in non-English languages. Full-text screening was conducted for the remaining 23 articles, resulting in the exclusion of 13 additional studies. Ultimately, 10 articles were included in the final analysis (Figure 2).

### 3.1. Pathophysiology of Acute Retinal Arterial Occlusion

The vascular anatomy of the retina includes the central retinal artery (CRA), a branch of the ophthalmic artery that supplies blood to the optic disc and the four quadrants of the retina. The outer retina receives additional perfusion from the choroidal circulation, which originates from the ciliary arteries. A cilioretinal artery is present in approximately 5–30% of individuals. Originating from the short posterior ciliary artery, the cilioretinal artery supplies blood to the macula. In cases of CRAO with cilioretinal artery sparing, central vision may be preserved due to maintained macular perfusion [1,3]. The most common cause of retinal artery occlusion is embolism, typically arising from atherosclerotic plaques in the carotid arteries or the heart. Emboli are primarily composed of cholesterol, calcific material, or fibrin. Thromboembolic vascular occlusion shares pathophysiological features with ischemic stroke [1,9]. The retina’s ability to recover depends on whether the embolus or thrombus is dislodged and on the retinal ischemic tolerance time—defined as the period before irreversible retinal damage occurs. If arterial occlusion is relieved within 90 min, full recovery of retinal function and visual acuity is possible. Partial recovery may still occur if reperfusion takes place within 240 min of occlusion onset [4,5,9,10].

### 3.2. Imaging Modalities in RAO

#### 3.2.1. Fluorescein Angiography (FA)

FA remains the gold standard diagnostic tool for visualizing retinal vessels and assessing blood flow in patients with RAO. It provides detailed information regarding vessel structure, patency, and caliber [7,14]. In cases of RAO, FA typically reveals normal choroidal circulation with delayed retinal arteriovenous filling. In later stages, staining or leakage of the optic disc and vessel walls may be observed. A complete absence of vascular filling on FA suggests ophthalmic artery occlusion rather than CRAO [4,12,18,19]. Despite its diagnostic value, FA has several limitations that restrict its routine clinical application. It is an invasive procedure requiring intravenous injection of an exogenous dye, which carries risks such as nausea, vomiting, allergic reactions, and, in rare cases, anaphylaxis or death. Furthermore, the procedure is time-consuming, typically requiring at least 15 min to complete [7,18]. Motion artifacts may also compromise FA image quality. Although FA offers valuable insights into retinal vascular structures, it is less effective in detecting deep ischemia or subtle alterations in the deep capillary plexus. These limitations underscore the need for alternative diagnostic methods that offer greater sensitivity, higher resolution, and reduced invasiveness [15,16,19].

#### 3.2.2. Optical Coherence Tomography

OCT in RAO typically demonstrates increased central macular thickness (CMT), hyperreflectivity of the inner retinal layers extending to the outer plexiform layer, and hyporeflectivity of the outer retinal layers due to signal attenuation from shadowing. These changes are direct consequences of retinal ischemia [4,13,15,17]. OCT alterations begin within the first few hours following occlusion and reach maximal expression within the initial days. Markedly increased reflectivity across all inner retinal layers—corresponding to the whitish retinal edema observed on funduscopy—has been consistently reported in cases of complete CRAO [15,16,19]. Furthermore, CMT measurements have shown significant correlation with best-corrected visual acuity (BCVA), suggesting the potential utility of OCT-derived metrics as prognostic indicators in RAO [6,12,16]. While OCT provides valuable structural insights, it cannot directly visualize blood vessels or assess retinal and choroidal blood flow parameters, thereby limiting its ability to evaluate the functional characteristics of ischemia in CRAO [6,14]. Additionally, OCT primarily enables longitudinal assessment of retinal layers but cannot distinguish between different types of retinal arterial occlusions or accurately delineate the extent of vascular obstruction and ischemia. As a result, establishing a definitive diagnosis of CRAO based solely on OCT imaging remains challenging [13,14,15].

#### 3.2.3. Optical Coherence Tomography Angiography

OCTA is a novel, non-invasive imaging modality that enables visualization of the retinal and choroidal vasculature without the need for exogenous dye injection [11,13,17]. It utilizes low-coherence interferometry combined with split-spectrum amplitude decorrelation angiography to detect motion contrast from flowing red blood cells, generating both three-dimensional and en face images of the vascular network. This technique allows for detailed visualization of retinal blood flow and capillary perfusion, making it valuable for the diagnosis, evaluation, and follow-up of retinal vascular diseases, including CRAO, diabetic retinopathy, choroidal neovascularization, age-related macular degeneration, and retinal vein occlusion [7,11,16,17]. OCTA provides quantitative data on the retinal vasculature, specifically assessing the superficial capillary plexus (SCP), deep capillary plexus (DCP), and choroidal vessels. This enables evaluation of vascular density and blood flow alterations [12,15,19]. OCTA can detect capillary network disruptions and identify zones of non-perfusion or abnormal vessel proliferation, making it an essential tool for assessing retinal ischemia and microvascular changes [13,14,17].

OCTA characteristics in CRAO include:

- SCP and DCP

**Figure 3:**
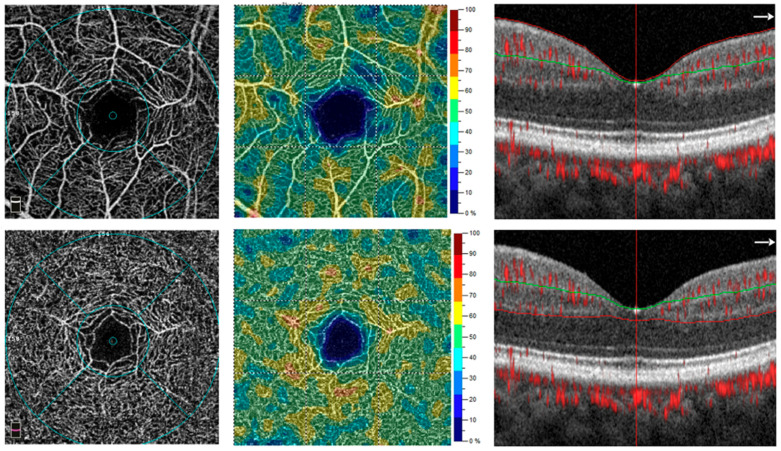
OCT-A image of a normal eye. The upper image represents the superficial capillary plexus (SCP), vessel density heat map of SCP, and B-scan centered on the fovea. The lower image represents the deep capillary plexus (DCP), vessel density heat map of DCP, and B-scan centered on the fovea. Courtesy of Yang et.al. [16].

**Figure 4:**
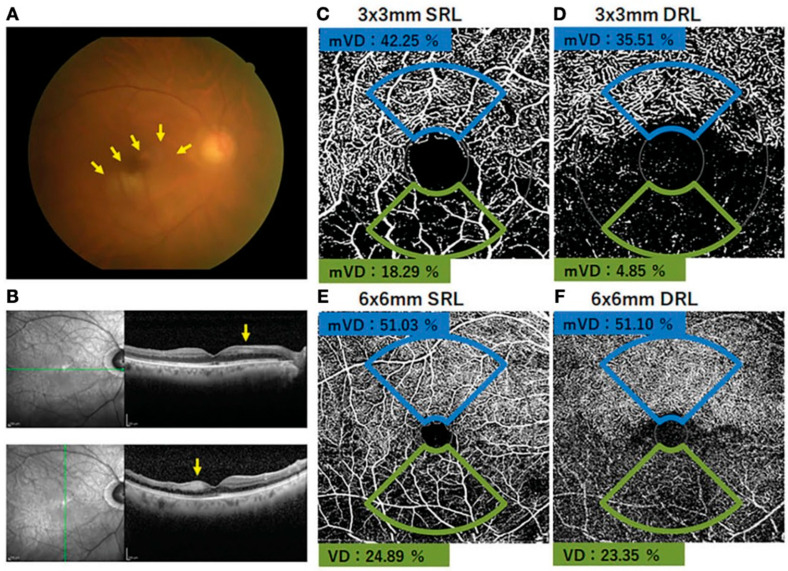
(**A**) Fundus photograph of the right eye with inferior branch retinal artery occlusion. The retinal whitening surrounding the occluded artery can be seen (arrows). The SD-OCT images show that the inner retinal layer is thicker and more hyperreflective on the affected area than on the normal area (arrows). (**B**) OCTA image shows the presence of flow in the superior artery and capillary bed, whereas no flow signal is evident in the inferior artery and capillary. (**C**–**F**) The macular vessel density (mVD) was significantly lower on the affected side than on the unaffected side for both the SRL and DRL in the 3 mm × 3 mm and 6 mm × 6 mm areas. Courtesy of Igawa et al., [13].

The vessel density of the SCP reflects the circulatory condition of the inner retina, which is primarily supplied by the retinal arterial system, while the vessel density of the DCP reflects the circulatory status of the outer retina, which is predominantly supplied by the choroidal circulation. [1,3,7]. The SCP is anatomically defined as the region between the vitreoretinal interface and the outer border of the ganglion cell layer, whereas the DCP lies between the inner border of the inner plexiform layer (IPL) and the outer border of the outer plexiform layer (OPL) (Figure 3) [13,14,17]. In CRAO, OCTA can reveal an ischemic pattern characterized by a disrupted capillary network and a darker background, indicative of absent or significantly reduced blood flow. The extent of involvement may vary, affecting the SCP, the DCP, or both [4,7,15,16,17,18,19]. Greater involvement of the SCP compared to the DCP in acute CRAO was observed by Igawa et al., who assessed vessel density in various macular regions of the affected eye. They reported a statistically significant reduction in SCP vessel density within the 3 mm concentric circle of the affected eye compared to the corresponding region in the unaffected eye (*p* = 0.022) (Figure 4). Conversely, no significant differences were observed in other quadrants of the SCP or in any regions of the DCP between the affected and unaffected eyes [13]. Consistent with the findings of Igawa et al., Lu et al. employed the nine Early Treatment Diabetic Retinopathy Study (ETDRS) subfields for macular segmentation. The average SCP vessel densities were reported as 0.36 ± 0.087 in the central foveal subfield, 0.36 ± 0.091 in the inner ring, 0.36 ± 0.090 in the outer ring, and 0.35 ± 0.084 for the full image, relative to the contralateral eye [12]. This finding may be explained by the anatomical and physiological features of the SCP. Located within the ganglion cell layer and directly supplied by the central retinal artery, the SCP is more immediately susceptible to perfusion deficits in RAO. Furthermore, the inner retina has a higher metabolic demand than deeper layers, increasing its vulnerability to ischemia. This observation aligns with broader evidence indicating SCP sensitivity in systemic microvascular compromise. For instance, a recent meta-analysis by Kazantzis et al. demonstrated significant enlargement of the FAZ in prediabetic patients compared to normoglycemic individuals, particularly in the SCP. This supports the hypothesis that the SCP may serve as an early biomarker of retinal microvascular dysfunction across a range of pathologies [21]. The vessel density in the DCP was found to be more reduced than in the SCP, with mean values of 40.54 ± 4.96 in RAO eyes, 45.88 ± 5.09 in fellow eyes, and 46.66 ± 5.51 in normal control eyes, demonstrating a statistically significant difference (*p* = 0.0011) [16]. Gong and colleagues further categorized CRAO into three types (A, B, and C) based on the severity of visual loss, extent of retinal edema, and delay in arterial blood flow observed on FA. They found that SCP vessel density was lowest in Type A CRAO, followed by Type B and then Type C, particularly at the central fovea (*p* = 0.001) and nasal parafovea (*p* = 0.001) when compared to healthy eyes. In contrast, Type B CRAO primarily affected the DCP and choroidal blood flow, whereas Type C CRAO showed significant impairment in the DCP, outer retinal layers, and choroidal vasculature [14]. Qualitatively, OCTA in RAO consistently reveals varying degrees of vascular nonperfusion in both the SCP and DCP [4,7,15,17,18,19]. A comprehensive summary of OCTA findings across the SCP and DCP is presented in Table 3. In examining imaging methodologies, we observed substantial heterogeneity in protocols across studies, including differences in scan size, software version, image processing algorithms, and segmentation approaches. These technical factors influence OCTA-derived metrics and likely account for variability in reported outcomes. For example, studies using swept-source OCTA—with improved choroidal penetration—tended to demonstrate more consistent DCP signals than those using spectral-domain platforms [12,13]. A consistent finding across several investigations was a more pronounced reduction in vessel density within the superficial plexus. Igawa et al. [13] reported a statistically significant decline in vessel density in the superficial layer within the 3 mm macular region, whereas no significant change was observed in the deep plexus. However, most studies presented divergent findings. Yang et al. [16] observed significant reductions in both plexuses when comparing RAO eyes to fellow and control eyes, and Bonini Filho et al. [17] described cases in which the deep plexus showed partial reperfusion despite persistent abnormalities in the superficial layer. These discrepancies likely reflect differences in disease severity, timing of imaging, and variation in OCTA platforms and image processing methods. Igawa et al. utilized a swept-source system with external thresholding methods, whereas Gong [14] and Li [7] employed spectral-domain systems with built-in automated segmentation. Differences in scan resolution, segmentation boundaries, and artifact correction likely contributed to inconsistencies in vessel density measurements, particularly between the superficial and deep layers. Methodological quality also varied across studies. As revealed by our ROBINS-I assessment, older studies such as those by Baumal [4] and Bonini Filho [17] were associated with serious risks of bias, including non-random sampling and incomplete data reporting. In contrast, more recent studies demonstrated greater transparency and improved methodological rigor.

- FAZ

Studies investigating the FAZ in CRAO have yielded mixed results. Several reports indicate reduced vessel density within a 300 μm radius around the FAZ, without significant differences in FAZ size between CRAO-affected eyes and fellow eyes [16,17]. In contrast, Jain and colleagues reported FAZ enlargement, with a mean size of 3.93 ± 0.84 mm^2^, along with persistent absence of macular reperfusion on follow-up OCTA imaging [15]. Similarly, Yang [16] and Li [7] found no measurable difference in FAZ size between affected and contralateral eyes, while Jain et al. [15] reported significant enlargement in affected eyes. These inconsistencies may be attributed to differences in segmentation accuracy, image resolution, and the lack of standardized definitions for FAZ measurements.

### 3.3. Peripapillary Area

The radial peripapillary capillary (RPC) system comprises distinct, long, straight, and rarely anastomosing vessels located within the nerve fiber layer. Originating from the peripapillary retinal arterioles, these capillaries are susceptible to various pathological conditions, notably glaucoma and ischemic retinal diseases [17]. In CRAO, the peripapillary region frequently exhibits reduced vessel density in both the SCP and DCP, indicating ischemic injury [7,16]. Additionally, OCTA findings in CRAO have shown either preservation or diffuse attenuation of the RPCs. In contrast, eyes with BRAO typically demonstrate focal attenuation of the RPCs, corresponding to the distribution of the affected arterial territory [4,17]. During the acute phase of CRAO, detectable flow is typically limited to the radial peripapillary capillaries. However, after approximately 8 weeks, partial re-establishment of flow within some retinal arterioles and relative preservation of the RPC network have been observed [17]. Moreover, OCTA can detect the development of collateral vessels around the optic nerve head in some CRAO cases, suggesting compensatory vascular responses [15].

### 3.4. CHOROID

Although less emphasized than retinal alterations, choroidal perfusion may also be reduced in RAO, likely secondary to compromised blood supply. The choroidal blood flow area in C-type CRAO (1.03) was significantly lower than that in A-type CRAO (1.81) (*p* = 0.018) [18]. In RAO eyes, the average choroidal volume was quantified as 0.22 ± 0.080 mm^3^ for the central foveal subfield, 0.43 ± 0.15 mm^3^ for the inner ring, 1.29 ± 0.39 mm^3^ for the outer ring, and 0.79 ± 0.24 mm^3^ for the entire image. In contralateral unaffected eyes, the corresponding average choroidal volumes were 0.21 ± 0.065 mm^3^ (central foveal subfield), 0.41 ± 0.13 mm^3^ (inner ring), 1.26 ± 0.36 mm^3^ (outer ring), and 0.77 ± 0.22 mm^3^ (entire image) [12]. The presence of a “cherry-red spot” in incomplete and subtotal CRAO is hypothesized to result from preserved choroidal blood flow despite concurrent ischemia affecting the superficial and deep retinal capillary plexuses [22]. Choroidal perfusion findings in RAO remain variable and limited. Gong et al. [14] observed a reduced choroidal flow area in more severe CRAO phenotypes, suggesting deeper vascular compromise; however, this remains underexplored due to the technical limitations of certain imaging systems.

### 3.5. Limitations of OCTA in RAO

Although OCTA is a highly valuable tool, it presents several limitations. Image quality is highly dependent on patient cooperation and the clarity of ocular media. In acute RAO, poor fixation due to vision loss can result in motion artifacts, including vessel duplication or distortion, which may obscure true pathology or mimic perfusion defects [4]. Moreover, the absence of established normative databases and variation across different OCTA platforms (e.g., segmentation software, scan size, resolution) poses additional challenges in comparing and standardizing OCTA parameters. Structural and optical reflectivity changes resulting from edema or atrophy in RAO may affect the accuracy of en face segmentation. Segmentation failures are common, especially in the presence of retinal architectural disruption. Automated layer detection algorithms may misclassify retinal boundaries, leading to inaccurate allocation of flow signals to the SCP or DCP [4,13,17,19]. Additionally, projection artifacts caused by superficial blood flow being projected onto deeper layers can further confound interpretation. These limitations reduce the reproducibility of OCTA metrics and underscore the importance of cautious interpretation, ideally alongside structural OCT and clinical correlation [4,17,19]. OCTA measurements can also be affected by factors such as axial length, refractive error, and age, although comparing symmetrical retinal areas within the same eye may help minimize these effects. Vascular identification in OCTA depends on the movement of blood cells within capillaries, and current algorithms may not detect very low flow. When flow velocity falls below the algorithm’s detection threshold—particularly in severely ischemic or non-perfused regions—capillaries may be erroneously interpreted as non-perfused. This limitation is especially relevant in acute RAO, where partial perfusion or sluggish flow may persist in marginally viable capillary beds but remain undetected, leading to false-negative assessments of perfusion loss [15,23]. Despite the increasing availability of OCTA in clinical ophthalmology, the absence of formalized clinician training in OCTA acquisition and interpretation remains a significant barrier to its effective use. Unlike traditional fluorescein angiography, which has well-established interpretation frameworks, OCTA introduces novel imaging biomarkers—such as non-perfusion zones, FAZ metrics, and layer-specific vessel density—that are not yet standardized across clinical practice. As a result, clinicians often face challenges in distinguishing true pathology from common artifacts, including motion blur, segmentation errors, projection artifacts, and shadowing caused by retinal edema or hemorrhage [24]. A standardized training curriculum should include comprehensive modules on OCTA physics, platform-specific software navigation, interpretation of en face and cross-sectional images, and differentiation between artifacts and clinically relevant findings. Furthermore, pattern recognition of vascular pathologies (e.g., flow voids in RAO vs. capillary dropout in diabetic retinopathy) must be emphasized. Comparative exercises involving fluorescein angiography and structural OCT would enhance multimodal interpretation skills, which are essential in complex cases. Interpretation of vessel density metrics also requires an understanding of device-specific segmentation boundaries and inter-individual anatomical variability. For example, SCP and DCP measurements may differ due to personal anatomical differences, necessitating contextualized analysis rather than strict reliance on numeric thresholds. Clinicians must also be aware of how image quality factors—such as signal strength index and axial length variation—can influence vessel density measurements [25].

### 3.6. Clinical Applications of OCTA in Acute RAO

Applying OCTA in routine clinical practice provides valuable information on changes in vessel density and perfusion density across the macular, foveal, parafoveal, and peripapillary regions, as well as variations in the FAZ area and choriocapillaris blood flow [16]. OCTA can quantify reductions in capillary plexus perfusion at both the superficial and deep layers in patients with RAO [7,16]. Its ability to detect hypoperfusion appears comparable to conventional FA, with improved contrast in visualizing small capillaries and identifying areas of capillary dropout [13,16]. Quantitative OCTA parameters—such as vessel density in the SCP and DCP, FAZ area, and choroidal perfusion—allow clinicians to assess the extent of microvascular compromise in a layer-specific and topographically precise manner. Notably, several studies have demonstrated that decreased vessel density in the macular SCP correlates with poorer baseline and final visual acuity, suggesting that this metric may serve as a functional prognostic indicator. For example, Lu et al. reported a statistically significant inverse correlation between SCP vessel density and visual acuity across multiple ETDRS subfields in RAO eyes, indicating that preserved superficial vascular architecture may predict better visual outcomes. In the acute management of RAO, OCTA enables rapid, high-resolution visualization of retinal perfusion status, facilitating immediate, evidence-based clinical decision-making. Layer-specific analysis of the SCP and DCP can be performed within minutes of presentation, allowing the prioritization of patients according to the extent and depth of ischemia. Preservation of DCP perfusion, or patchy SCP flow, suggests the presence of viable retinal tissue and supports urgent initiation of reperfusion measures—such as anterior chamber paracentesis, ocular massage, or intraocular pressure-lowering medications—within the narrow therapeutic window. Conversely, OCTA evidence of complete non-perfusion in both plexuses, particularly when accompanied by choriocapillaris compromise, may indicate irreversible damage. In such cases, clinical priorities may shift toward systemic evaluation for embolic sources, modification of vascular risk factors, and prevention of fellow-eye involvement, rather than aggressive acute intervention [12,14]. Furthermore, OCTA can differentiate between complete and incomplete occlusions, as well as between central and branch involvement, based on the distribution of capillary non-perfusion. Its ability to detect subclinical ischemia—often not visible on conventional funduscopy—further enhances its value in the acute phase. OCTA also facilitates early identification of patients at risk of progressive nonperfusion areas, allowing for timely referral to stroke units and systemic evaluation. Moreover, it enables longitudinal monitoring of vascular recovery or deterioration without the risks associated with dye-based angiography. Serial OCTA imaging can reveal dynamic changes such as reperfusion, collateral vessel formation, or expansion of ischemic zones—key findings that guide follow-up intensity and systemic vascular risk management. In contrast to FA, which is invasive and unsuitable for frequent repetition, OCTA permits high-resolution, serial monitoring of microvascular status with minimal patient burden [7,16].

### 3.7. Monitoring and Follow-Up

OCTA provides an effective tool for monitoring and evaluating treatment response in acute CRAO, particularly in detecting the restoration of blood flow following early intervention. Given its non-invasive nature and ability to be performed repeatedly, OCTA is well-suited for assessing the effects of therapeutic interventions such as hyperbaric oxygen therapy, ocular massage, or intra-arterial thrombolysis. Changes in perfusion density or FAZ configuration over time can help evaluate treatment efficacy and monitor disease progression. Unlike FA, which cannot be repeated at every visit due to its invasive nature and associated risks, OCTA offers a safe, repeatable imaging modality that enables longitudinal assessment without additional patient burden [13,17].

### 3.8. The Applications of Artificial Intelligence

Artificial intelligence (AI) has revolutionized numerous fields of medicine, particularly those that rely heavily on imaging modalities for diagnosis and disease evaluation [26]. The development of AI-based technologies—including machine learning (ML), deep learning (DL), support vector machines (SVMs), and convolutional neural networks (CNNs)—has significantly influenced ophthalmology. These innovations have transformed the processing and interpretation of data from imaging modalities such as fundus photography, ultrasonography, CT, MRI, OCT, and especially OCTA [27]. RAO represents a promising area for AI-driven diagnostic support. Although research remains in its early stages, techniques such as CNNs show potential in diagnosing RAO using OCTA by automatically detecting areas of capillary non-perfusion, evaluating vessel density reduction, and identifying FAZ enlargement. DL models may further assist in distinguishing RAO from other retinal vascular conditions, offering systematic analysis with greater sensitivity and consistency than manual interpretation [26,27].

### 3.9. Future Direction

Recent advancements in OCTA have introduced novel quantitative biomarkers that may offer improved sensitivity and diagnostic utility. Among these, vessel length density (VLD) and fractal dimension (FD) have been used to more accurately characterize the retinal microvasculature. VLD quantifies the total length of perfused vessels within a given area and is less influenced by large vessel dominance compared to conventional vessel density metrics. FD, on the other hand, measures the complexity and branching behavior of the vascular network, reflecting both developmental and pathophysiological changes. In a large-scale analysis of healthy subjects, Wang et al. [28] demonstrated that both VLD and FD varied significantly across retinal regions and demographic groups, underscoring their physiological relevance. Although that study focused on normative data, it highlights the potential applicability of these metrics in disease states such as RAO. Incorporating VLD and FD into OCTA-based RAO assessments may improve the sensitivity of ischemia detection, enable more refined phenotyping, and enhance comparability across studies using diverse imaging protocols.

### 3.10. Limitations

While meta-analytic synthesis is a valuable approach for generating statistically robust conclusions and estimating pooled effect sizes, its validity depends on a high degree of homogeneity in study design, patient populations, outcome definitions, and measurement tools. In this review, substantial methodological and technical heterogeneity among the included studies precluded the feasibility of a formal meta-analysis. Although forest and funnel plots are valuable for visually summarizing study-level effects and assessing publication bias, these could not be generated due to pronounced variability in OCTA protocols, segmentation methods, and outcome reporting, as well as the limited number of studies with directly comparable metrics. Future OCTA research should prioritize standardized acquisition protocols, analytical criteria, and reporting frameworks to enable quantitative synthesis, thereby enhancing the reproducibility and clinical applicability of systematic review findings.

## 4. Conclusions

Acute RAO is a sight-threatening emergency characterized by microvascular ischemic changes, which can be effectively evaluated using OCTA. This systematic review highlights OCTA as a valuable non-invasive tool for diagnosing and assessing RAO. OCTA consistently reveals characteristic perfusion deficits in both the superficial and deep retinal plexuses, and its quantitative parameters correlate with visual outcomes, indicating prognostic value. Despite its advantages, limitations remain—including motion artifacts and the absence of standardized normative data. Future efforts should prioritize the standardization of OCTA protocols and analytical methods to enhance its clinical utility and improve patient care.

## Figures and Tables

**Table 1:** The terms used in the search for each website.

S. No	Databases	Search String	No. of Records
1	Web of Science	TS = (“Optical coherence tomography angiography” OR “OCTA”) AND TS = (“Retinal arterial occlusion” OR “Central retinal artery occlusion” OR “Branch retinal artery occlusion” OR “RAO” OR “CRAO” OR “BRAO”)	48
2	Medline	TS = (“Optical coherence tomography angiography” OR “OCTA”) AND TS = (“Retinal arterial occlusion” OR “Central retinal artery occlusion” OR “Branch retinal artery occlusion” OR “RAO” OR “CRAO” OR “BRAO”)	63
3	EMBASE	(“optical coherence tomography angiography”/exp OR “optical coherence tomography angiography”:ti,ab,kw OR “octa”:ti,ab,kw) AND (“retinal artery occlusion”/exp OR “retinal arterial occlusion”:ti,ab,kw OR “central retinal artery occlusion”:ti,ab,kw OR “branch retinal artery occlusion”:ti,ab,kw OR “rao:ti,ab,kw” OR “crao:ti,ab,kw” OR “brao:ti,ab,kw”)	161
4	Scopus	(TITLE-ABS-KEY (“Optical coherence tomography angiography” OR “OCTA”)) AND (TITLE-ABS-KEY (“Retinal arterial occlusion” OR “Central retinal artery occlusion” OR “Branch retinal artery occlusion” OR “RAO” OR “CRAO” OR “BRAO”)) AND (LIMIT-TO (DOCTYPE, “ar”)) AND (LIMIT-TO (LANGUAGE, “English”))	105

**Table 2:** Risk of bias assessment.

Study	Overall Risk of Bias	Bias Due to Confounding	Bias in Selection of Participants into the Study	Bias in Classification of Interventions/Exposures	Bias Due to Deviations from Intended Interventions/Exposures	Bias Due to Missing Data	Bias in Measurement of Outcomes	Bias in Selection of the Reported Result
[4]	Serious	Moderate: Small sample, unaddressed heterogeneity	Serious: Non-random enrollment, small sample size	Low: Clear definitions for RAO	Not applicable	Serious: High exclusion rate, inconsistent measurement	Moderate: Manual segmentation, acknowledged artifacts	Low: No explicit selective reporting
[13]	Moderate	Low: Within-subject comparison.	Moderate: Convenience sample, very small size	Low: Clear classification of affected/unaffected	Not applicable	Moderate: Exclusions for image quality, unquantified	Moderate: Acknowledged image processing artifacts	Low: No explicit selective reporting
[7]	Moderate	Moderate: Lack of adjustment for systemic confounders	Moderate: Retrospective, single-center, convenience	Low: Clear definitions of groups	Not applicable	Low: Based on info, but total exclusions are unknown.	Low: Automated/standardized measurements.	Low: No explicit selective reporting
[15]	Moderate	Moderate: Potential unmeasured confounders	Moderate: Retrospective, single-center, reliance on OCT	Low: Robust classification of ILMD	Not applicable	Moderate: Missing data for imaging, unquantified exclusions	Low: Standardized outcomes, dual-reader for quantitative	Low: No explicit selective reporting
[12]	Moderate	Low: Within-subject comparison.	Moderate: Convenience sample, image quality exclusions	Low: Clear classification of RAO	Not applicable	Moderate: Significant exclusions for NPA analysis	Low: Automated/dual-reader for measurements.	Low: No explicit selective reporting
[18]	Moderate	Moderate: Potential unmeasured confounder	Moderate: Retrospective, single-center, convenience	Low: Clear classification of PAMM	Not applicable	Low: No explicit missing data reported.	Low: Automated/standardized measurements.	Low: No explicit selective reporting
[17]	Serious	Moderate: Small sample, unaddressed heterogeneity	Serious: “Offered enrollment,” high exclusion rate	Low: Clear definitions for RAO	Not applicable	Serious: High exclusion/inconsistent measurement	Moderate: Manual segmentation, acknowledged artifacts	Low: No explicit selective reporting
[19]	Serious	Low: Within-subject comparison.	Moderate: Convenience sample, small size	Low: Clear CRAO diagnosis	Not applicable	Serious: Significant missing OCTA data	Moderate: Qualitative assessments subjective	Low: No explicit selective reporting
[14]	Moderate	Moderate: Potential unadjusted systemic confounders	Moderate: Retrospective, single-center, convenience	Low: Clear NA-CRAO type classification	Not applicable	Moderate: Exclusions for image quality/FFA.	Low: Automated/standardized measurements.	Low: No explicit selective reporting
[16]	Moderate	Moderate: Unaddressed systemic confounders	Moderate: Retrospective, single-center, convenience	Low: Clear RAO diagnosis	Not applicable	Moderate: Exclusions for image quality, missing data	Low: Automated/standardized measurements.	Low: No explicit selective reporting

**Table 3:** Summary of OCTA findings.

Study (First Author, Year)	OCTA Machine Used	Software Used for Analysis	Findings in Superficial Capillary Plexus (SCP)	Findings in Deep Capillary Plexus (DCP)
[13]	SS-OCTA (PLEX Elite 9000, Carl Zeiss Meditec, Inc. Jena, Germany).	Built-in segmentation software, Otsu analysis with ImageJ software of the National Institutes of Health (Bethesda, MD, USA).	Significantly lower vessel density in the affected side for a 3 mm concentric circle. No significant difference for a 6 mm concentric circle.	No significant difference in vessel density between affected and unaffected sides for a 3 mm concentric circle.
[4]	(RTVue XR Avanti, Optovue Inc., Fremont, CA, USA).	AngioVue SD-OCTA software within the commercially available RTVue XR Avanti device (Optovue Inc., Fremont, CA, USA).	Decreased vascular perfusion. In BRAO, a larger area of decreased vascular perfusion was observed in 75% of eyes compared to DCP.	Decreased vascular perfusion. In acute CRAO, equal areas of decreased vascular perfusion to the SCP. In BRAO, one eye showed a wider area of decreased vascular perfusion, and partial restoration of perfusion in chronic RAO.
[7]	RTVue XR (Optovue, Inc., Fremont, CA, USA).	RTVue-XR version 2017.1.0.155.	Significantly reduced vessel density in all areas except the fovea in RAO eyes compared to fellow and normal control eyes. No significant difference between fellow eyes and normal control eyes.	Significantly reduced vessel density in all areas except the fovea in RAO eyes compared to fellow and normal control eyes. Reduced considerably in fellow eyes compared to normal control eyes in all areas except the fovea.
[15]	Topcon TRITON 3D PLUS Version 10.19 (Topcon, Tokyo, Japan).	Not explicitly stated, likely integrated with the Topcon system.	Showed attenuation of both arteries and veins with significant macular flow void areas.	Showed pruning of both arteries and veins with significant macular flow void areas.
[19]	AngioVue XR Avanti system (Optovue Inc., Fremont, CA, USA).	Not explicitly stated, likely integrated with the AngioVue system.	Disruption observed. More compromised than DCP. Marked disruption with decreased vascular perfusion.	Disruption observed. Less compromised than SCP. Marked disruption with decreased vascular perfusion.
[12]	Zeiss Plex Elite 9000 WF SS-OCTA (Carl Zeiss Meditec, Inc., Jena, Germany).	Advanced Retina Imaging (ARI) Network (Zeiss Portal v5.4-1206).	Decreased vascular perfusion. Statistically significant inverse correlation between SCP vessel density and vision in all subfields for RAO eyes. No correlation in contralateral eyes.	Less involved than SCP. Inverse correlation between DCP vessel density and vision in all subfields for RAO eyes, but not statistically significant. No correlation in contralateral eyes.
[18]	RTVue-XR Avanti (Optovue, Inc., Fremont, CA, USA).	Vue software within the commercially available RTVue XR Avanti device (Optovue Inc., Fremont, CA, USA).	Significantly decreased density in the non-PAMM group with broken, branch-like changes. No significant difference in overall superficial vascular density between PAMM and no-PAMM groups.	Reduced considerably the density in the non-PAMM group with broken branch-like changes. No significant difference in overall deep vascular density between PAMM and no-PAMM groups.
[14]	RTVue-XR Avanti.	Not explicitly stated, likely integrated with the RTVue-XR Avanti system.	Vessel density significantly decreased in Type A patients compared to controls. Lowest in Type A at the central fovea and the nasal parafovea compared to B and C types. No significant difference in other regions among types.	Vessel density significantly decreased in all three types of NA-CRAO patients compared to the control group. C type decreased the most (except fovea), but no significant differences were observed among the three types. Common damage targets in all CRAO types.
[16]	RTVue-XR Avanti (Optovue Inc. Fremont, CA, USA).	AngioVue Analytics, RTVue-XR version 2017.1.0.155 software.	Significant decrease in vessel density in RAO eyes compared with fellow eyes and normal control eyes. Significantly lower in RAO fellow eyes than in normal control eyes. Reduced in the affected hemifield in BRAO.	Significant decrease in vessel density in RAO eyes compared with fellow eyes and normal control eyes. No significant difference between fellow eyes and normal control eyes. Reduced in the affected hemifield in BRAO.
[17]	Prototype Angio Vue SD-OCTA software (RTVue XR Avanti; Optovue, Inc.).	AngioVue spectral-domain OCTA software within the commercially available RTVue XR Avanti device (Optovue Inc, Fremont, CA, USA).	Decreased vascular perfusion in all affected eyes. In CRAO with cilioretinal sparing, focal restoration of deep capillary plexus perfusion, while SCP perfusion was abnormal. In BRAO eyes, 75% showed focal restoration of deep capillary plexus perfusion where SCP was abnormal.	Decreased vascular perfusion in all affected eyes. In CRAO, equal areas of decreased vascular perfusion as in SCP. In CRAO with cilioretinal sparing and BRAO eyes (75%), focal restoration of perfusion was observed in regions where SCP was abnormal, and partial restoration of perfusion was observed in chronic RAO.

## Abbreviations

The following abbreviations are used in this manuscript:

SJ	Saud Aljohani
AS	Abdulazizi Alshehri
